# Low-grade infections as a possible cause of arthrofibrosis after total knee arthroplasty

**DOI:** 10.1186/s13037-018-0181-1

**Published:** 2019-01-10

**Authors:** C. Brückner, E. Straube, I. Petersen, S. Sachse, P. Keller, F. Layher, G. Matziolis, U. Spiegl, D. Zajonz, M. Edel, A. Roth

**Affiliations:** 1 Orthopaedic Professorship of the University Hospital Jena, Orthopaedic Department of the Waldkliniken Eisenberg, Eisenberg, Germany; 2 Institute of Medical Microbiology, Friedrich-Schiller-University Jena, Jena, Germany; 3 Institute of Pathology, Friedrich-Schiller-University Jena, Jena, Germany; 4 Institute of Pathology, SRH Waldklinikum Gera, Gera, Germany; 5 Institute of Medical Microbiology, University of Zurich, Zurich, Switzerland; 6 Department of Orthopaedics, Traumatology and Plastic Surgery, University Hospital Leipzig, Leipzig, Germany; 7 ZESBO – Center for research on musculoskeletal systems, Leipzig, Germany; 8 Klinik und Poliklinik für Orthopädie, Unfallchirurgie und Plastische Chirurgie, Bereich Endoprothetik/Orthopädie, Universitätsklinikum Leipzig AöR, Liebigstraße 20, 04103 Leipzig, Germany

**Keywords:** Total knee arthroplasty, Arthrofibrosis, PCR

## Abstract

**Purpose:**

Arthrofibrosis after total knee arthroplasty represents a considerable burden for the patient and a therapeutic challenge for the practitioner. One possible cause discussed in the literature is a low-grade infection. This hypothesis should be examined within the scope of this retrospective study.

**Patients and methods:**

Nineteen patients with clinical symptoms of arthrofibrosis after primary total knee arthroplasty were examined between January, 1999 and January, 2012. Incorrect positioning was radiologically ruled out. All patients were examined clinically (score of Freeman as well as Blauth and Jäger), radiologically (component and leg alignment, patella height according to Insall and Salvati), microbiologically (culture-based procedures), molecular biologically (PCR) and histologically in the course of an open revision of the prosthesis.

**Results:**

According to the score of Freeman et al. (1977), a highly significant improvement in pain (*p* = 0.007) and in the overall score (*p* = 0.003) was shown. The knee joint mobility did not change significantly (*p* = 0.795). PCR was negative in 17 patients. One patient showed a PCR-positive result of the synovial membrane for Corynebacterium spp., while Staphylococcus warneri was detected in the culture. Another patient had a positive result of synovia PCR for Enterococcus cecorum as well as Corynebacterium spp. However, this culture was sterile. In 16 patient samples, no bacterial growth was detectable. Two samples were not evaluable. The main histopathological findings were synovialitis and fibrosis.

**Conclusion:**

The hypothesis of low-grade-infection-induced arthrofibrosis after total knee arthroplasty could not be confirmed in this study. However, based on this small study population the conclusion needs to be confirmed by new and larger studies, ideally prospectively designed including a control group.

## Introduction

The causes of postoperative pain after a total knee arthroplasty, which is accompanied by limited mobility, often remain unclear. The clinical pathology corresponds to stiff-knee or arthrofibrosis, although a precise definition of the disease is still lacking. Incorrect implant positioning and instabilities can be possible causes [1]. A hypothesis for the development of arthrofibrosis is a low-grade infection [2, 3].

Arthrofibrosis is described as a progressive and fibrous process within a joint, often associated with inflammation [4, 5]. Up to the present day, numerous hypotheses with the same fundamental idea exist, which is based on a hypoxia of the synovialis caused by a circulatory disorder [5]. This is thought to induce a distinctly increased synthesis of fibrotic material in the sense of “pathological wound healing” or “disturbed remodelling” [6]. At the beginning it is typically localized, but over time arthrofibrosis can spread throughout the entire joint. If the practitioner finds a cause for this condition, such as an incorrect implantation or an insufficient postoperative mobilization, a revision operation is conceivable. In most patients, however, it is difficult to accurately identify the pathogenesis that causes arthrofibrosis.

A favoured hypothesis of the development of arthrofibrosis is infection. Joints treated with an implant are more susceptible to infections than joints without an implant [2, 3]. While high-grade joint infections often exhibit a clear clinical and microbiological indication of inflammation, low-grade infections usually provide no distinct evidence. Thus, this kind of infection still presents a particular challenge to today’s clinical practices and diagnostics.

This retrospective study examines the hypothesis of low-grade-infection-induced arthrofibrosis after primary total knee arthroplasty. In order to confirm this assumption, samples were taken from the synovia as well as synovial membrane during revision operation. Subsequently, the samples were examined for bacteria using conventional microbiological analytical methods (microscopy, pathogen culture) and 16S-rRNA-PCR as supplementary molecular genetic diagnostic procedure. Moreover, it should be determined whether the microbiological findings correlate with the histopathology of arthrofibrosis. If the hypothesis of low-grade infection as the source of arthrofibrosis is confirmed, it will optimize preoperative diagnosis and treatment for patients with this disorder.

## Material and methods

The study was approved by the ethics committee of the Friedrich-Schiller-University Jena (No. 3409–03/12).

In consideration of previously defined inclusion and exclusion criteria (Table 1), 19 patients with clinically confirmed arthrofibrosis after primary total knee arthroplasty (01/1999–01/2012) were re-examined within the scope of a revision operation (01/2010–01/2012). Intraoperatively samples of the synovia (one sample) and synovial membrane (three samples) were taken and examined to rule out an infection using conventional microbiological (microscopy, pathogen culture) and molecular biological methods (16S-rRNA-PCR). In cases without measurable DNA concentrations, a GAPDH-PCR was performed. To confirm the clinical suspicion of arthrofibrosis, three further samples of the synovial membrane were taken for the histopathological examination.

**Table 1:** Inclusion−/exclusion criteria of the study

inclusion criteria	exclusion criteria
- persistent painful limitation of motion after TKA - high degree of psychological strain, restricted quality of life - informed consent concerning the study design	- high-grade-infection - incorrect implantation of the TKA - drug abuse - temporary immobilization after the revision

The anamnesis included location and time of primary total knee arthroplasty, retention time of the implant up to revision, invasive or surgical interventions prior to and after total knee arthroplasty as well as secondary diseases.

Before revision surgery and three months after revision, the following clinical parameters were collected: effusion, swelling, hyperthermia, instabilities, retropatellar symptoms of discomfort and the range of motion (extension/flexion) according to the Neutral Zero method. During the same time interval, the clinical scores pursuant to Freeman et al. [7, 8] as well as Blauth and Jäger [4, 8] were evaluated. While pain intensity, ability to walk and range of motion (Table 2) are assessed by the score of Freeman et al., the classification of knee joint stiffness (Table 3) is evaluated using the score of Blauth and Jäger.

**Table 2:** Score of Freeman et al. [7]

Pain	None	50	“acceptable”
	Mild (an “occasional twinge”, not a spontaneous complaint, does not require analgesia, does not limit function)	40	“acceptable”
	Moderate (may require analgesia but does not limit function)	15	
	Severe (any other pain)	0	
Ability to walk	Outdoors, 30 minutes or more	20	“acceptable”
	Outdoors, 0 - 30 minutes	15	“acceptable”
	Indoors	5	
	Unable	0	
Range of motion	80 °	30	“acceptable”
	60 ° - 79 °	20	
	30 ° - 59 °	5	
	0 ° - 29 °	0	
If “acceptable” in all three categories add:		10	
Acceptable Result:	Overall assessment	95 - 110 points	
	Pain	40 - 50 points	
	Function	15 - 20 points	
	Movement	30 points

**Table 3:** Score of Blauth and Jäger [4]

grade I	Range of motion at least 90 °
grade II	Range of motion 60°-90°
grade III	Range of motion 30°-60 °
grade IV	Range of motion at least range 30 °

Radiologically, the valgus- and varus-angle were determined via an image of the entire lower extremity. The slope and the patella height according to Insall and Salvati [9] were specified in the lateral beam path.

In all patients, open revision and arthrolysis followed after a closed anaesthetic mobilisation via a medial parapatellar approach. An intraoperative single-shot antibiotic treatment was performed after sample extraction in all patients.

In addition to the descriptive data presentation [mean value (MV), standard deviation (σ), minimum (min), maximum (max)] the statistical evaluation (SPSS version 19) includes the analysis of the changes resulting from the revision. In this context, the mobility and clinical scores pursuant to Freeman et al. [7, 8] as well as Blauth and Jäger [4, 8] are evaluated by means of the Wilcoxon test. A cross-table and the chi-square test according to Pearson were used to check the frequency distribution of the score of Blauth and Jäger [4, 8]. The significance level of all statistical tests was set to *p* ≤ 0.05.

## Results

**Fig. 1:**
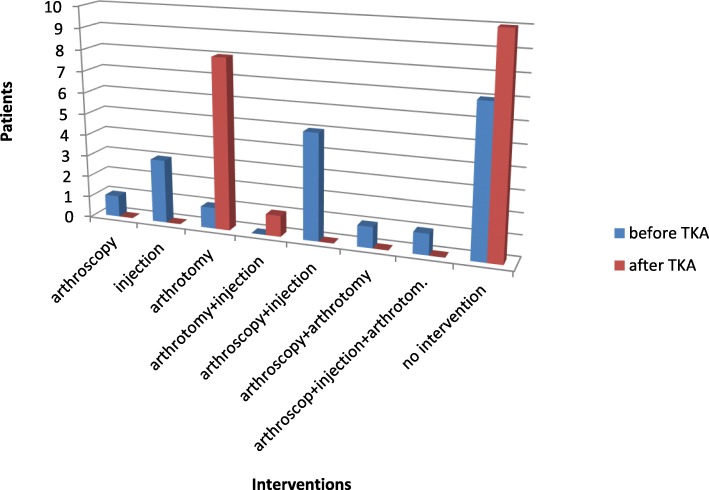
invasive interventions before/after total knee arthroplasty

Nineteen patients (11 men, 8 women) with an average age of 66.37 years (σ = 8.34 years) were included in the study. All patients suffered from relevant knee pain at and limited range of movement after initial total knee arthroplasty. With reference to the previously defined exclusion criteria, two patients were excluded from the clinical follow-up and the score collection. Overall, 11 left and eight right knee joints were affected. Thirteen of these patients received a total knee endoprosthesis in-house and six patients externally. In 12 patients, at least one arthroscopic procedure was performed prior to primary endoprosthesis implantation. The average time interval between primary implant surgery and revision was 3.39 years (σ = 2.76 years). An arthrotomy was performed in nine patients during this period (Fig. 1).

Five patients showed obesity as a secondary disease. Additionally, it was proven that two of them were affected by hyperuricemia and one by solitary hyperuricemia. Two more patients suffered from neurological diseases (1x Parkinson’s disease, 1x infantile cerebral palsy with diplegia and spasticity).

**Fig. 2:**
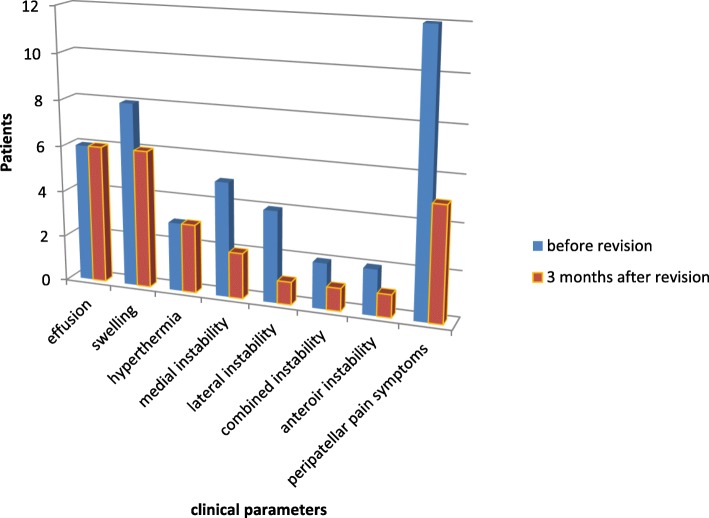
clinical parameters before/after revision

The evaluation of the clinical examination parameters prior to revision surgery (19 patients) revealed as cardinal symptoms peripatellar pain symptoms, instability and swelling followed by effusion and hyperthermia. After revision surgery (17 patients) effusion, swelling, and peripatellar pain represented the main symptoms (Fig. 2).

**Fig. 3:**
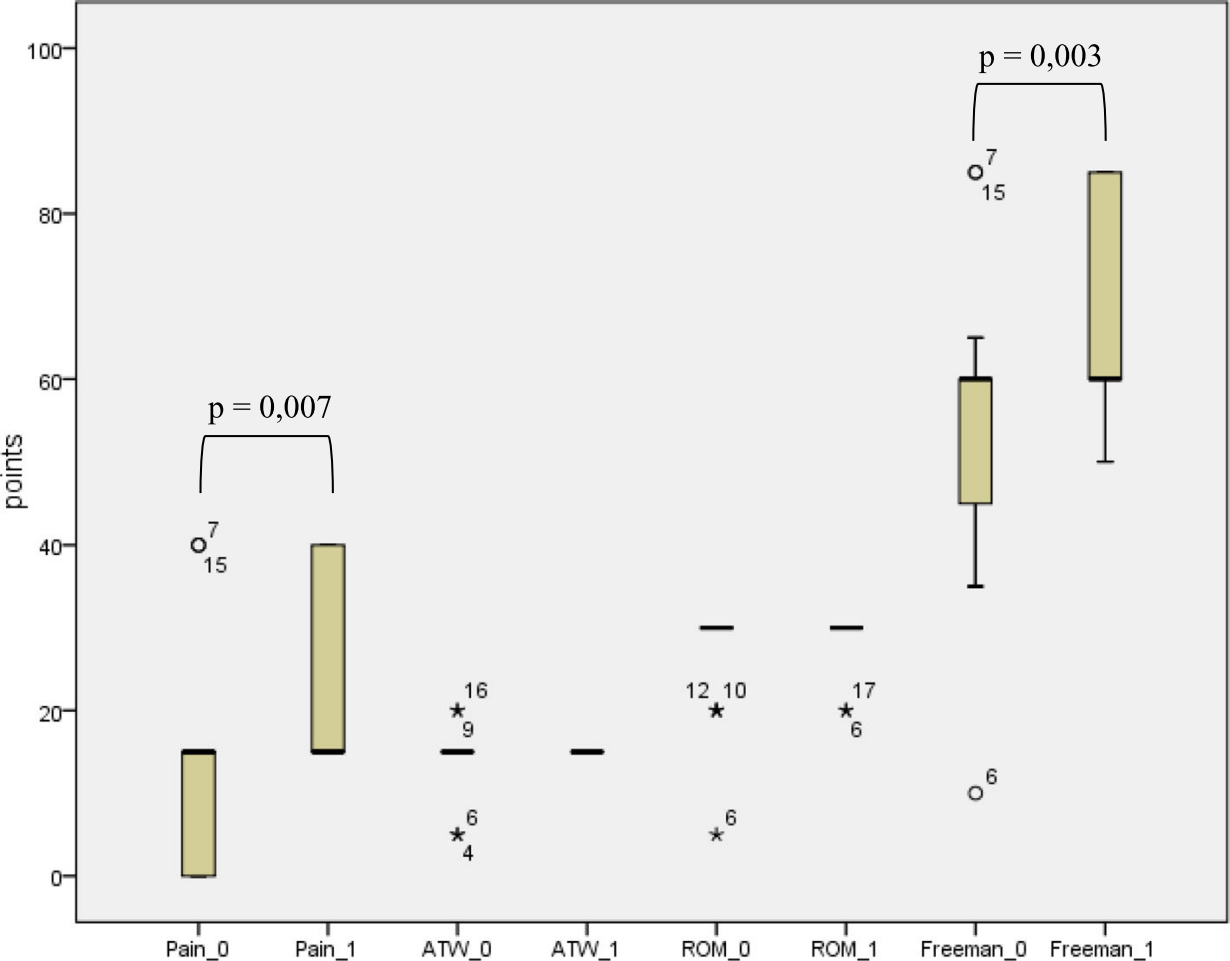
score of Freeman et al. [7] before/after revision

**Fig. 4:**
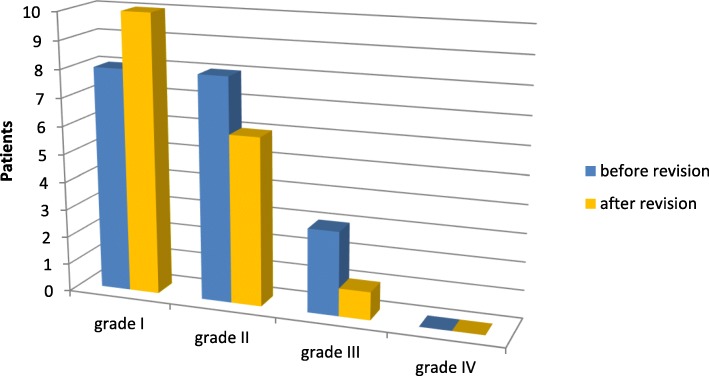
score of Blauth and Jäger [4]

All patients had a preoperative limitation of mobility related to flexion, extension or both. Neither flexion, nor the extension deficit and overall range of motion showed any significant improvement three months after revision (Table 4). With regard to the score of Freeman et al. [7, 8] there was a highly significant reduction of pain (*p* = 0.007) and an improvement in the overall score (*p* = 0.003). Walking ability (ATW) (*p* = 0.458) and mobility of the knee joint (ROM) (*p* = 0.157) were not significantly altered (Fig. 3). According to the score of Blauth and Jäger [4, 8] no significant changes of the stiffness of the affected knee could be detected (*p* = 0.708) (Fig. 4).

**Table 4:** Flexion/extension deficit and ROM before/after revision

	Minimum	Maximum	Mean value	Standard deviation
Flexion before revision	40 °	130 °	91.76 °	20.38 °
Flexion after revision	65 °	125 °	92.06 °	13.70 °
Extension deficit before revision	0 °	20 °	2.65 °	5.04 °
Extension deficit after revision	-5 °	10 °	1.76 °	3.93 °
Range of motion before revision	35 °	130 °	89.12 °	22.52 °
Range of motion after revision	55 °	125 °	90.29 °	15.26 °

The radiological examination showed an average tibiofemoral alignment of 6.7 ° (σ = 3.2 °), whereas the tibial slope averaged 7.2 ° (σ = 4.1 °). Concerning the score of Insall and Salvati [9], eight patients had a normally positioned patella (LT/LP = 1.03), eight patients a patella alta (LT/LP > 1.15) and three patients a patella baja (LT/LP < 0.75) (normal range 0.8–1.04).

**Fig. 5:**
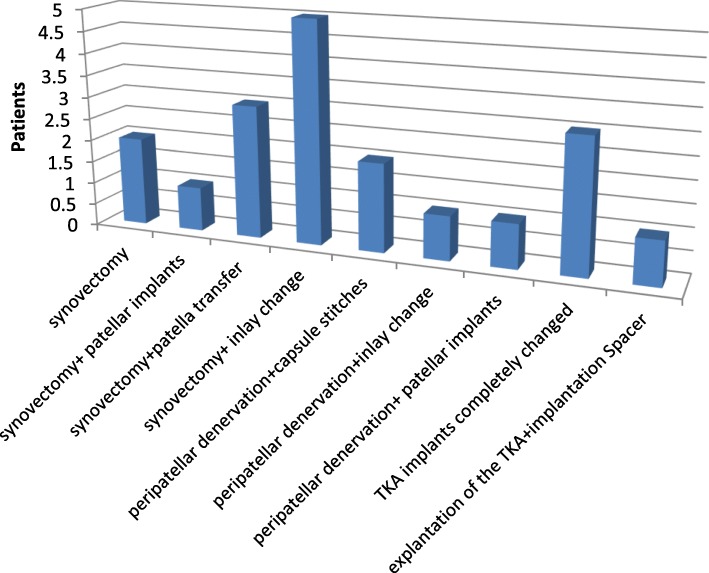
additional operative interventions during revision

In addition to closed manipulation and open arthrolysis, the most frequent interventions were synovectomy (*n* = 11), followed by exchange of the inlay (*n* = 6), and peripatellar denervation (*n* = 4) (Fig. 5). Furthermore, a spacer was implanted in one patient with a positive culture, followed by a reimplantation as a two stage procedure. In three cases a complete replacement of the prosthesis was performed due to pronounced ligamentous instability.

PCR was negative in 17 patients. One patient showed a PCR-positive result of *Corynebacterium spp.* at the synovial membrane*.* Additionally, *Staphylococcus warneri* was found in the culture. Another patient had a positive result of synovia PCR for *Enterococcus cecorum* as well as *Corynebacterium spp.* However, all cultures of this patient remained sterile. No bacterial growth was detectable in all further samples (16 patients). Two samples could not been evaluated (Table 5).

**Table 5:** Microbiological and molecular biological results

Patients	Culture Synovia	Culture Synovial Membrane	PCR Synovia	PCR Synovial Membrane
15	sterile	sterile	negative	negative
1	sterile	sterile	positive *Enterococcus cecorum* *Corynebacterium spp*.	negative
1	positive *Staphylococcus warneri*	sterile	negative	positive *Corynebacterium spp*.
2	not evaluable	not evaluable	negative	negative

The preoperative laboratory values showed no significant increase (CRP: mean 9.20 mg/l, min. 0.3, max. 44.9; leucocytes: mean 10.6 Gpt/l, min. 4.6, max. 10.6). For the patients tested positive in the PCR, they were for CRP at 15.7 resp. 1.5 mmol/l and for the leucocytes at 9.7 resp. 4.8 Gpt/l.

**Fig. 6:**
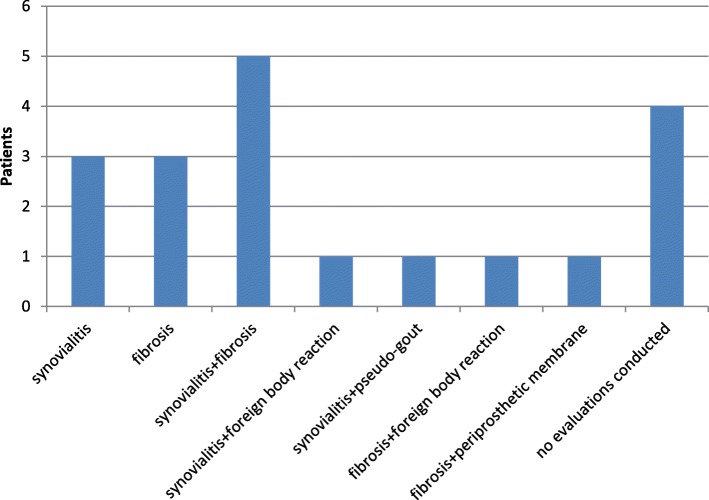
histopathological results

The main histopathological results were synovialitis, fibrosis and synovialitis with fibrosis. All the other histologies exhibited at least one of the latter two characteristics. In five cases, the term “arthrofibrosis” was used in the finding (Fig. 6). Moreover, no granulocytic elements indicating a low-grade infection were found.

## Discussion

Arthrofibrosis is usually an inflammatory, progressive fibrous process. After total knee arthroplasty, it occurs with a probability of up to 10% [5, 10–12]. In our patients collective, it was conspicuous that arthroscopy had been performed in 12 out of 19 patients (63.2%) before primary knee arthroplasty. Particularly arthroscopy represents a significant risk factor for the development of arthrofibrosis [13, 14]. In the investigated patient collective, secondary diseases favouring arthrofibrosis [1, 15] were found in the form of obesity (26.3%), obesity with hyperuricemia (10.5%), hyperuricemia (5.3%) and pre-existing neurological diseases (10.5%). However, no significant accumulation of secondary diseases could be detected.

In addition to instability, effusion, swelling and hyperthermia, peripatellar pain was the main clinical symptom [16–18]. Prior surgery, 63% of our patients complained of retropatellar pain, whereas this percentage dropped to 29% after revision surgery. One reason for this is the high contact pressure of the patella against the femoral component [19]. Unfortunately, no significant improvement in the range of motion could be achieved through revision surgery. However, a significant and relevant reduction in pain was visible. The authors consider this as the most important benefit in patients with sufficient knee function for daily living.

Radiologically, incorrect endoprosthesis positioning could be ruled out. Moreover, our results show that patella alta (eight patients) occurred more frequently than a patella baja, which is a strong risk factor for the development of peripatellar pain symptoms after total knee arthroplasty [18, 20].

In part, arthroscopy is recommended as a first-line therapy [21, 22]. However, the period between primary implantation and revision surgery should be between three and six months, maximum one year [23]. In these 19 patients, this period was up to three years and therefore arthrotomy was consistently chosen [24–26].

The inflammatory parameters (CRP and leucocytes) did not help us to diagnose an active periprosthetic infection, particular in patients with intraarticular bacterial detection. In the PCR of the synovial membrane of one patient gram-positive bacterium *Corynebacterium spp*., typical bacteria of the skin, was found [27]. Its culture delivered *Staphylococcus warneri*, conspicuous for joint infections [28, 29]. The synovia PCR of a second patient was positive for *Enterococcus cecorum* and *Corynebacterium spp*., which are again part of the normal skin and mucous membrane flora [27]. However, the culture of this patient was negative.

Divergent results between culture and PCR can be explained by the limited sensitivity of PCR to different pathogen concentrations of individual bacteria. Furthermore, this could also be caused by an unknown outpatient antibiotic treatment. In addition, a migration of pathogen DNA by macrophages and granulocytes via the bloodstream is possible [30–36]. Moreover, false-positive results in the culture due to contamination, e.g. during sampling, transport and processing in the laboratory, cannot be completely ruled out [37, 38].

The classic microbiological methods (microscopy and pathogen culture) can remain false-negative in spite of an existing infection due to insufficient bacterium load, the presence of a highly variable pathogen species with delayed growth cycles or due to an antibiotic therapy. Especially in the case of “difficult-to-treat” bacteria like “small colony variants” (SCV), it is sometimes necessary to cultivate them over a long period of time [15, 39]. Moreover, it can be difficult to unmask the individual pathogens in a mixed polymicrobial flora [15]. Therefore, multiple inspections of the samples appear to be useful [40].

Morgenstern et al. were able to demonstrate that the results of PCR were essentially comparable to those of the culture in the diagnosis of periprosthetic infections [41]. Here, PCR was more suitable for the detection of low virulence bacteria such as *Cutibacterium spp*. and coagulase-negative Staphylococci. However, it shows the fundamental suitability of both methods. In the case of negative cultures, the performance of a PCR can be appropriate and expedient in a justified individual case, despite the additional time and cost [31].

The histopathological examination results of our study depicted inflammatory processes in the form of synovialitis and fibrosis. However, there were no granulocytic elements indicating a low-grade infection. Abdul et al. histologically described a dramatic tissue remodelling, increased collagen deposition and increased (myo)fibroblast staining in tissue from revision total knee arthroplasty [54]. Therefore, conventional histologies are not sufficient to define histopathological changes as an “arthrofibrosis”.

The present study has some limitations. First of all, its retrospective design has to be mentioned. The number of patients included was low, which leads to an unpowered study and risk of type II statistical error. Minor criteria for periprosthetic infections were not known at the time of taking the samples and therefore were not considered in the present study [55]. Furthermore, it would have been desirable to integrate a control group without arthrofibrosis into the study in order to draw comparisons and conclusions. Several approaches of the same samples are perspectively recommended for PCR. Moreover, a follow-up study with a higher number of cases, which may be multi-sited, could contribute to a verification of the results.

Arthrofibrosis is a progressive process of joint fibrosis accompanied by inflammatory reactions. There was a universal definition and consensus in international panels of experts in 2016 [42]. These authors defined a post-operative fibrosis as the limited range of movement in flexion and/or extension, that is not attributable to an osseous or prosthetic block to movement from malaligned, malpositioned or incorrectly sized components, metal hardware, ligament reconstruction, infection (septic arthritis), pain, chronic regional pain syndrome (CRPS) or other specific causes, but due to soft-tissue fibrosis that was not present pre-operatively. From the authors’ point of view, pain represents one of the most essential symptoms. The cause of arthrofibrosis after total knee endoprosthesis is multifactorial [43–53].

If the hypothesis of a low-grade-infection-induced arthrofibrosis had been supported, a decisive optimization of the pre-operative diagnosis and subsequent therapy would perspectively have been possible. However, our study of 19 patients showed that a low-grade infection was not the cause of arthrofibrosis. For this reason, the hypothesis must be rejected. However, based on the limited patients included over a time period of three years, which extrapolates to five or six patients a year prospectively designed studies such as multi-sited studies including a control group are warranted to support this conclusion.

## Conclusion

The hypothesis of low-grade-infection-induced arthrofibrosis after total knee arthroplasty could not be confirmed in this study.

## Abbreviations

ATW: Ability to walk
CRP: C-reactive protein
CRPS: Chronic regional pain syndrome
DNA: Deoxyribonucleic acid
GAPDH: Glyceraldehyde 3-phosphate dehydrogenase
LP: Length of the patella/largest diagonal diameter of the patella
LT: Length of the patella tendon
MV: Mean value
PCR: Polymerase chain reaction
ROM: Range of motion
rRNA: Ribosomal ribonucleic acid
SCV: Small colony variants
